# Biochemical determinants of the IGFBP‐3–hyaluronan interaction

**DOI:** 10.1002/2211-5463.12919

**Published:** 2020-07-22

**Authors:** Sadaf Dorandish, Jonathan Devos, Bradley Clegg, Deanna Price, Robert Muterspaugh, Jeffrey Guthrie, Deborah L. Heyl, Hedeel Guy Evans

**Affiliations:** ^1^ Department of Chemistry Eastern Michigan University Ypsilanti MI USA

**Keywords:** CD44, humanin, hyaluronan, IGFBP‐3, kinetics, peptide

## Abstract

IGFBP‐3, the most abundant IGFBP and the main carrier of insulin‐like growth factor I (IGF‐I) in the circulation, can bind IGF‐1 with high affinity, which attenuates IGF/IGF‐IR interactions, thereby resulting in antiproliferative effects. The C‐terminal domain of insulin‐like growth factor‐binding protein‐3 (IGFBP‐3) is known to contain an 18‐basic amino acid motif capable of interacting with either humanin (HN) or hyaluronan (HA). We previously showed that the 18‐amino acid IGFBP‐3 peptide is capable of binding either HA or HN with comparable affinities to the full‐length IGFBP‐3 protein and that IGFBP‐3 can compete with the HA receptor, CD44, for binding HA. Blocking the interaction between HA and CD44 reduced viability of A549 human lung cancer cells. In this study, we set out to better characterize IGFBP‐3‐HA interactions. We show that both stereochemistry and amino acid identity are important determinants of the interaction between the IGFBP‐3 peptide and HA and for the peptide's ability to exert its cytotoxic effects. Binding of IGFBP‐3 to either HA or HN was unaffected by glycosylation or reduction of IGFBP‐3, suggesting that the basic 18‐amino acid residue sequence of IGFBP‐3 remains accessible for interaction with either HN or HA upon glycosylation or reduction of the full‐length protein. Removing N‐linked oligosaccharides from CD44 increased its ability to compete with IGFBP‐3 for binding HA, while reduction of CD44 rendered the protein relatively ineffective at blocking IGFBP‐3‐HA interactions. We conclude that both deglycosylation and disulfide bond formation are important for CD44 to compete with IGFBP‐3 for binding HA.

## Graphical abstract

Binding of IGFBP‐3 either to humanin (HN) or to the glycosaminoglycan, hyaluronan (HA), is not affected by either glycosylation or reduction. Glycosylated and reduced CD44 is less able than de‐N‐glycosylated and oxidized CD44 to compete with IGFBP‐3 for binding HA.

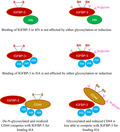

**Abbreviations**
HA: hyaluronan
HABD: HA‐binding domain
HN: humanin
IGFBP‐3: insulin‐like growth factor‐binding protein‐3
IGF‐I: insulin‐like growth factor I
NSCLC: non‐small‐cell lung cancer

Insulin‐like growth factor‐binding protein 3 (IGFBP‐3) is a protein that belongs to a family consisting of six IGF‐binding proteins that share highly conserved structures consisting of three distinct (N‐terminal, linker, and C‐terminal) domains [1, 2, 3, 4, 5, 6]. IGFBP‐3, the most abundant IGFBP and the main carrier of insulin‐like growth factor I (IGF‐I) in the circulation, can bind IGF‐1 with high affinity, attenuating IGF/IGF‐IR interactions, resulting in antiproliferative effects [1, 4, 6]. IGFBP‐3 is also known to operate via different mechanisms to regulate cell survival independently of the IGF/IGF‐IR axis [6, 7, 8, 9]. Lower expression of IGFBP‐3 [10] in lung cancer is known to be associated with poor diagnosis in patients with stage I non‐small‐cell lung cancer (NSCLC) [11, 12, 13, 14, 15]. An inverse relationship between plasma or serum levels of IGFBP‐3 and lung cancer risk has been previously shown [1, 6, 16]. Increased expression of IGFBP‐3 corresponded with diminished survival of human lung cancer cells [17]. Crosstalk between IGFBP‐3 and the GFBP‐related protein, IGFBP‐7, that binds IGF‐1 with low affinity is being increasingly recognized [18]. IGFBP‐7 has been found to act as a tumor suppressor in lung cancer with its expression being high in healthy lung tissue but downregulated in lung cancer largely due to DNA hypermethylation [18].

Hyaluronan (HA) is a anionic, nonsulfated glycosaminoglycan [19, 20, 21, 22] polymer consisting of the disaccharide sequence (d‐glucuronic acid and d‐*N*‐acetylglucosamine). It is abundant extracellularly as a chief component of the extracellular matrix [20, 23, 24]. Via interactions with its binding proteins, HA has been implicated in the remodeling of the matrix that occurs during the pathogenesis of several human diseases [24, 25, 26]. The steady‐state levels of HA are known to be low in most normal tissues, while production of HA and its accumulation in the tumor parenchyma are known to occur in certain types of cancer such as lung cancer and are associated with poor clinical outcomes, enhancing proliferation and metastasis [20, 24, 25, 27, 28].

CD44, known to bind HA, is a type I transmembrane glycoprotein, encoded by a single gene [19, 20, 21, 28, 29, 30]. From the N terminus to the C terminus, the structure of CD44 consists of a globular HA‐binding domain (HABD), a stalk domain, a single‐pass transmembrane domain, and a cytoplasmic domain [20, 21, 28, 29, 31]. Found on the extracellular side of the cell membrane are the domains located N‐terminal to the transmembrane domain, including HABD, while those C‐terminal to the transmembrane domain are found intracellularly [20, 21, 23, 28, 30, 31]. Recombinant expression of the HABD was shown to retain its ability to bind HA as a globular water‐soluble protein [32]. Alternative splicing is known to generate many different variant isoforms (CD44v) containing variable patterns of amino acid insertion into the stalk domain, with the standard CD44 (CD44s) being the smallest [33, 34]. The HA‐binding motif is composed of residues 32–123 in the N‐terminal domain of CD44, a region common to both CD44s and CD44v isoforms [19]. CD44s is the predominant isoform expressed in human lung cancer cell lines [35] that include A549 used in this study [36]. Binding of HA to CD44, its main receptor, is thought to promote cell survival pathways [26, 37, 38, 39, 40].

The strength of the HA‐CD44 interaction is known to be modulated by post‐translational modifications that include attachment of carbohydrates to O‐ and N‐linked glycosylation sites on the extracellular portion that includes HABD [20, 21, 33, 37, 41]. Inhibition of CD44 N‐glycosylation was found to enhance HA binding [42]. The role of both N‐ and O‐glycosylation is conflicting and depending on the cell lines and methods used, and HA‐CD44 binding has been reported to be positively or negatively regulated by glycosylation [19, 21, 28, 42]. Force spectroscopy was employed previously to show that inhibition of O‐glycosylation did not significantly alter the frequency of binding of either CD44v or CD44s to HA [19]. However, N‐linked glycosylation was shown to positively correlate with HA‐CD44 binding in lung epithelium‐derived tumor cells expressing CD44s, which include the cells used in this study [21, 43]. When the N‐glycans on HABD are attached to sialic acid residues, however, HA‐CD44 binding was inhibited which was suggested to be possibly due to interaction of sialic acids with basic amino acids that might otherwise bind HA [44].

CD44 activity is also regulated by the extracellular redox environment through the secretion of thiol reductase and protein disulfide isomerase [45], enzymes with emerging roles in cancer [46]. Kinetic trapping and binding experiments using CD44 proteins found that a labile disulfide bond formed between Cys77 and Cys97 in CD44 stabilizes the HA‐binding groove [47]. Reduction of this bond inhibited HA‐CD44 interaction *in vitro*, and pretreatment with reducing agents blocked the ability of cells to adhere to HA‐coated surfaces [47].

Previously, we found that IGFBP‐3 binds HA via amino acid residues 215–232 in the C‐terminal region of the protein (^215^‐KKGFYKKKQCRPSKGRKR‐^232^), blocking HA interactions with CD44 and reducing viability of A549 human lung cancer cells [48]. These results are in support of findings from previous reports showing that this region of IGFBP‐3 is able to bind certain glycosaminoglycans, including HA [1, 6, 49, 50, 51]. We also showed that using the anti‐CD44 antibody (5F12) to block HA‐CD44 binding in combination with IGFBP‐3 did not have an additive negative effect on cell viability, suggesting that IGFBP‐3 operates via a mechanism that depends on HA‐CD44 interactions to exert its cytotoxic effects on cell survival [48].

Humanin (HN), first discovered by the Nishimoto laboratory [52, 53], is a mitochondrial‐derived peptide known to bind with high affinity and specificity to the 18‐amino acid residue (215–232) heparin‐binding domain of IGFBP‐3 [54] that also binds HA. HN was shown to interact with the C‐terminal domain of IGFBP‐3, blocking IGFBP‐3‐induced cell death, without affecting or competing with binding of IGF‐I to IGFBP‐3 [55]. We also found that HA binding to the 215–232 segment of IGFBP‐3 renders it inaccessible for binding to HN and that either HA or HN could bind to this IGFBP‐3 segment, but not simultaneously [48]. Accumulating evidence suggests that HN has wide neuro‐ and cytoprotective activities against different types of stress and a broad range of disease models [56, 57, 58]. In response to cellular stress, HN needs to be secreted to exhibit diverse extracellular signaling functions and broad cytoprotective effects in various diseases [53, 54]. It is known to be regulated by growth hormone and IGF‐I and, in turn, regulates IGF‐I likely through binding to the C‐terminal region of IGFBP‐3 [55]. Previously, we showed that HN binds IGFBP‐3 and blocks its interaction with importin‐β1 *in vitro*, providing a probable function for HN in regulating the nuclear translocation of IGFBP‐3 [59]. More recently, we found that aggregation of amyloid‐β induced by acetylcholinesterase is blocked by the presence of HN [60]. Binding of IGFBP‐3 to HN, however, sequesters HN away from amyloid‐β, increasing its aggregation [60].

IGFBP‐3 is also known to be N‐glycosylated [5, 51, 61] although the biological significance of this modification is not clear [1, 49, 62]. The N‐linked glycosylation‐specific sites have been identified in the amino‐terminal and linker domains, and the affinity for the IGFs was shown to be identical for both the glycosylated and nonglycosylated forms of IGFBP‐3 [1, 63]. Moreover, the lack of glycosylation either had no effect or slightly increased the affinity of IGFBP‐3 for the cell membrane [1, 63, 64]. IGFBP‐3 contains a total of nine conserved disulfide bonds with six located in the N‐terminal domain and three in the C‐terminal domain [1, 5]. While the biological significance of these bonds is unclear at present, the N‐terminal and C‐terminal domains are not thought to be linked by disulfide linkages, with intradomain disulfide bonds being more likely to occur than interdomain disulfide linkages formed between cysteines in the N‐terminal and C‐terminal domains [65]. In this study, we examine factors that might modulate the interactions between HA and IGFBP‐3.

## Materials and methods

### Materials

Most of the material used was purchased as we previously reported [48, 60, 66]. Phosphate‐buffered saline (PBS), streptavidin‐conjugated horseradish peroxidase (HRP), phenylmethylsulfonyl fluoride (PMSF), TCEP‐HCl, *N*‐ethylmaleimide, and HA‐biotin (B1557) were purchased from Sigma‐Aldrich (Burlington, MA, USA). PNGase F was from New England Biolabs (Ipswich, MA, USA). Recombinant human IGFBP‐3 protein (YCP1009, UniProt accession ID: P17936) was purchased from Speed BioSystems (Gaithersburg, MD, USA). Human recombinant CD44 protein (12211‐H08H) was purchased from Sino Biological (Wayne, PA, USA). Nonglycosylated recombinant IGFBP‐3 (MBS142177) was from MyBioSource (San Diego, CA, USA). HN (018‐26) and biotin‐HN (B‐018‐26, UniProt accession ID: Q8IVG9) were purchased from Phoenix Pharmaceuticals (Burlingame, CA, USA). CD44 antibody (5F12) (MA5‐12394), mouse IgG isotype control (mIgG), ultra 3,3′,5,5′‐tetramethylbenzidine (TMB)‐ELISA substrate solution, Halt Protease and Phosphatase Inhibitor Cocktail, and Nunc MaxiSorpTM 96‐well flat‐bottom plates were from Thermo Fisher. The SuperSignal West Pico Luminol (Chemiluminescence) Reagent and BCA Protein Assay Kit were from Pierce (Dallas, TX, USA).

### Solid‐phase peptide synthesis and purification

We synthesized the 18‐amino acid residue (215–232) heparin‐binding domain of IGFBP‐3 in the C‐terminal region of the protein [54] that also binds HA, the corresponding analogous peptides in the C‐terminal domains of IGFBP‐1, IGFBP‐2, and IGFBP‐4‐6 (Table 1), along with the IGFBP‐3 mutant (K228AR230A), as a negative control, since we previously showed that it completely lacks the ability to bind HA [66]. Fluorenylmethyloxycarbonyl (Fmoc)‐protected l‐amino acids and *O*‐benzotriazolyl‐*N*,*N*,*N*′,*N*′‐tetramethyluronium hexafluorophosphate (HBTU), used to synthesize the peptides in this study, were purchased from Anaspec Inc (Fremont, CA, USA). The D‐IGFBP‐3 peptide was synthesized in the same manner using Fmoc‐protected amino acids in the D‐configuration purchased from Combi Blocks (San Diego, CA, USA). Dichloromethane (DCM) was purchased from Acros Organics (Fair Lawn, NJ, USA). Dimethylformamide (DMF) and HPLC‐grade acetonitrile (ACN) were from VWR. Piperidine, triisopropylsilane (TIS), diethyl ether, ethanol, phenol, and trifluoroacetic acid (TFA) were purchased from Sigma‐Aldrich. Rink amide MBHA resin was purchased from Sigma‐Aldrich.

**Table 1:**
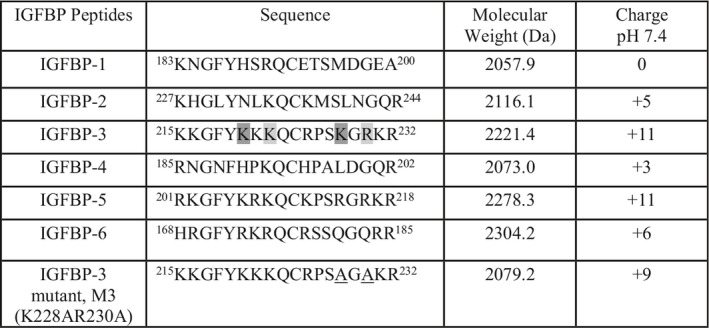
Sequences of the synthetic peptides, determined molecular weights, and charges. The first and last amino acid residues of the overlapping HA‐binding sequences in IGFBP‐3 are shaded [66] and the mutated amino acids are underlined.

Peptides were synthesized as described previously [66] on a 0.1 mmole scale on methylbenzhydrylamine (MBHA) resin using a Protein Technologies PS3 Peptide Synthesizer (Tucson, AZ, USA). A tert‐butyl group was used as side‐chain protection for Asp, Glu, Ser, and Tyr; t‐butyloxycarbonyl (Boc) was utilized for the Lys side‐chain amine; Asn, Gln, His, and Cys were protected with a trityl (Trt) group; and the guanidine group of Arg was protected with a 2,2,5,7,8‐pentamethyl‐chroman‐6‐sulfonyl (Pmc) moiety. Amino acids were coupled using HBTU as the activating agent, in fourfold excess to resin sites. The Fmoc group was removed after each coupling using 20% piperidine in DMF. Upon completion, the resin was washed with DMF, ethanol, and DCM and then dried under vacuum. Peptides were cleaved from the resin with simultaneous protecting group removal using TFA (8.8 mL), distilled water (0.5 mL), phenol (0.5 mL), and TIS (0.2 mL), with stirring at RT for 2 h. After filtration of the resin, cold diethyl ether was used to precipitate the peptides from solution. The precipitate was then isolated by filtration, redissolved in 35% ACN/H_2_O, and lyophilized. RP‐HPLC was used to purify the peptides using a Phenomenex C18 column (25 cm × 2.2 cm), with a solvent system of 0.1% TFA in water (solvent A) and 0.1% TFA in ACN (solvent B), with a 2‐h gradient of 10–50% solvent B at 10 mL·min^−1^. Final purity was determined at 220 nm by analytical RP‐HPLC using a Phenomenex C18 column (25 cm × 4.6 mm). Peptide molecular weights were confirmed by paper spray ionization mass spectrometry. For assays, peptides were dissolved in 1% DMSO in pH 7.4 PBS buffer, to a final concentration of 1 mg·mL^−1^.

### Liposome dye leakage

The liposome dye leakage assay was performed as we reported previously [67] to determine whether the peptides had any membrane‐damaging properties. Lipids were purchased from Avanti Polar Lipids (Alabaster, AL, USA), and carboxyfluorescein was purchased from Sigma‐Aldrich. Carboxyfluorescein‐encapsulated liposome vesicles were created and tested as previously described [67]. Briefly, a 5 mg mixture of 3 : 1 1‐palmitoyl‐2‐oleoyl‐glycero‐3‐phosphocholine (POPC, a zwitterionic lipid)/1‐Palmitoyl‐2‐oleoyl‐sn‐glycero‐3‐(phospho‐rac‐(1‐glycerol)) (POPG, a negatively charged lipid) was weighed and dissolved in 2 mL chloroform; the chloroform was evaporated using nitrogen, leaving a thin lipid film which was dried in a vacuum desiccator overnight. After addition of 500 µL of 30 mm carboxyfluorescein dye in sodium phosphate buffer (pH 7.5), the tubes were vortexed and incubated for 1 h at RT. Vesicles were formed by five successive freeze–thaw cycles in liquid nitrogen. This solution was then extruded through a polycarbonate filter (pore size 100 nm) 21 times using a mini‐extruder from Avanti Polar Lipids (fitted with two 0.5‐mL Hamilton gastight syringes). Nonencapsulated dye was removed from the vesicles using a Sephadex G50 gel exclusion column. Stock solutions of peptides (70 μm) were prepared in PBS (pH 7.4) with 5% DMSO; controls also contained 5% DMSO/PBS, which was predetermined not to cause dye leakage. Final peptide concentration in the wells also containing PBS and vesicles was 20 µm. After a 10‐min period, fluorescence values of the samples in 96‐well plates were measured by a spectrofluorometer (filter set to 485 nm excitation and 528 nm emission). Triton X‐100 detergent (10% v/v in PBS) was used as the positive control for determination of 100% leakage, while the negative control was 5% DMSO in PBS.

Dye leakage was calculated by the equation below where *F*_solvent_ is the fluorescence of the negative control (no peptide). Values reported are the average of triplicate runs.

$$\text{Percentage}\;\text{of}\;\text{dye}\;\text{leakage}=\frac{F‐F_{\text{solvent}}}{F_{\text{detergent}}‐F_{\text{solvent}}}\times 100.$$

### Cell culture

HFL1 (ATCC CCL‐153) normal, nontransformed, and nontumorigenic human fibroblast cell line and the human NSCLC cell line, A549 (ATCC CCL‐185), were purchased from the American Type Culture Collection (ATCC, Manassas, VA, USA). Cells were seeded as we reported earlier [48, 60, 66] in 5 mL HyClone Dulbecco's modified Eagle's medium/nutrient mixture F‐12 (DMEM/F12) (GE Healthcare Life Sciences, Pittsburgh, PA, USA), supplemented with 10% Fetalgro bovine growth serum (FBS; RMBIO, Missoula, MT, USA), 50 U·mL^−1^ penicillin, and 50 U·mL^−1^ streptomycin (Invitrogen Life Technologies, Carlsbad, CA, USA) in 25‐cm^2^ tissue culture flasks, and allowed to grow overnight in an incubator at 37 °C, 95% humidity, and 5% CO_2_. Cells were counted with a hemocytometer after trypan blue staining.

### ELISA

ELISA was conducted as we reported previously [48, 60, 66, 68]. Nunc MaxiSorp 96‐well flat‐bottom plates (Thermo Fisher, Waltham, MA, USA) were coated with samples as indicated. The plates were incubated overnight at 4 °C on a shaker to allow binding of the samples to the plate wells. After the incubation, the wells were washed 4× with TBST, filled with 400 µL blocking buffer (110 mm KCl, 5 mm NaHCO_3_, 5 mm MgCl_2_, 1 mm EGTA, 0.1 mm CaCl_2_, 20 mm HEPES, 1% BSA, pH 7.4), and incubated overnight at 4 °C on a shaker. The wells were then washed 4× with TBST, and 100 µL of sample at the desired concentration was added to each well before incubating overnight at 4 °C on a shaker. TBST was then used to wash the wells 4× before proceeding in one of two ways: (a) Biotinylated samples were analyzed by adding 100 µL streptavidin–HRP conjugate in TBST (1 : 2500 dilution) to the samples before incubating for 3 h at RT on a shaker, or (b) samples without biotin were analyzed by adding 100 µL TBST containing the primary antibody at the manufacturer's recommendation and incubating for 3 h at RT on a shaker before washing 4× with TBST. The secondary antibody in 100 µL TBST was then added to the samples following the manufacturer's recommendation and incubated for 1 h at RT on a shaker. Plates containing either biotinylated or nonbiotinylated samples were then washed 5× with TBST followed by the addition of 100 µL TMB resulting in a blue color change. The reaction was stopped with 100 µL 2 m H_2_SO_4_ after incubating at RT for 0.5–15 min, resulting in a yellow color change that was measured by absorbance at 450 nm. To monitor nonspecific binding, negative control wells on the plates included, for example, bound IGFBP‐3 then adding all components, streptavidin–horseradish peroxidase and TMB, but without addition of biotin‐HN. Some wells were coated with 2.5, 10, 50, 100, 500, and 5000 nm biotin‐HN or biotin‐HA to allow conversion of the OD measurements to concentrations of bound material. Before analysis, the OD from the data was corrected for nonspecific binding by subtracting the mean background absorbance for the negative controls. Typically, in control wells incubated on each plate, the background binding is about 10–15% of the maximum binding seen with addition of biotin‐HN or biotin‐HA or antibodies. Statistical analysis was determined by the graphpad prism 8.4.2 software (San Diego, CA, USA). Data were expressed as the mean ± SD. Three to five independent experiments were carried out in triplicate for each assay condition.

### TCEP‐HCl reduction and deglycosylation

Glycosylated recombinant His‐tagged IGFBP‐3 (Speed BioSystems) and nonglycosylated recombinant IGFBP‐3 (MyBioSource, San Diego, CA, USA) were used as reported earlier [69]. Glycosylated human CD44 recombinant protein was purchased from Sino Biological (Wayne, PA, USA). To cleave N‐linked glycans, the proteins were treated with PNGase F for 4 h at 37 °C under nondenaturing conditions according to the manufacturer's specification. To reduce the proteins and following previous protocols [47], the purified proteins were reduced with TCEP‐HCl (2.5 mm in PBS/1%BSA for 20 min at 4 °C) and then alkylated by 5 mm NEM for 30 min at 4 °C, to permanently block disulfide bond formation. As control, nonreduced samples treated with only 5 mm NEM were used.

### MTT assay

The MTT reduction assay (Sigma‐Aldrich), used to measure cell viability, was employed as we reported earlier [48, 60, 66, 67]. Cells were seeded in 96‐well plates as indicated in 200 μL 10% FBS‐supplemented media per well and maintained overnight at 95% humidity and 5% CO_2_. After an overnight incubation, the media was replaced with 200 μL serum‐free media and the cells were then allowed to incubate for a further 24, 48, or 72 h. The final concentration of DMSO in each well never exceeded 0.1%. Following treatment, the cells were incubated for 4 h with MTT (0.5 mg·mL^−1^) in the dark. The media was carefully removed, and DMSO (100 μL) was added to dissolve the formazan crystals. The absorbance was measured at 570 nm in a plate reader. Untreated cells or wells containing only DMSO and media were used as a positive and negative control, respectively. Statistical analysis was conducted using graphpad prism version 8.4.2 for Windows. Significant values were considered at *P* < 0.05 and more significant values at *P* < 0.01, compared with the control.

### Statistical analysis

Each experiment in this study was performed in triplicate and repeated a minimum of three times. Statistical values are expressed as the mean ± standard deviation (SD) as we reported previously [48, 60, 66]. To evaluate the statistical differences, the Mann–Whitney or Kruskal–Wallis (ANOVA) test was used. All the statistical tests were two‐sided, and a *P* value of <0.05 was considered statistically significant in all cases. graphpad prism (GraphPad Software, 8.4.2) was used for the statistical analysis.

## Results and Discussion

### Membranolytic activity is unlikely to be the main mechanism employed by the IGFBP‐3 peptide

Except for IGFBP‐1, the IGFBP peptides possess sequences with stretches of cationic amino acids and positive charge with IGFBP‐3 and IGFBP‐5 having the highest positive charge of +11 at pH 7.4 (Table 1). In order to examine whether the synthetic IGFBP peptides possess physiochemical features that enable them to target and disrupt lipids in cell membranes, we used an *in vitro* assay as we previously reported [67], to evaluate their membrane lytic properties.

**Fig. 1:**
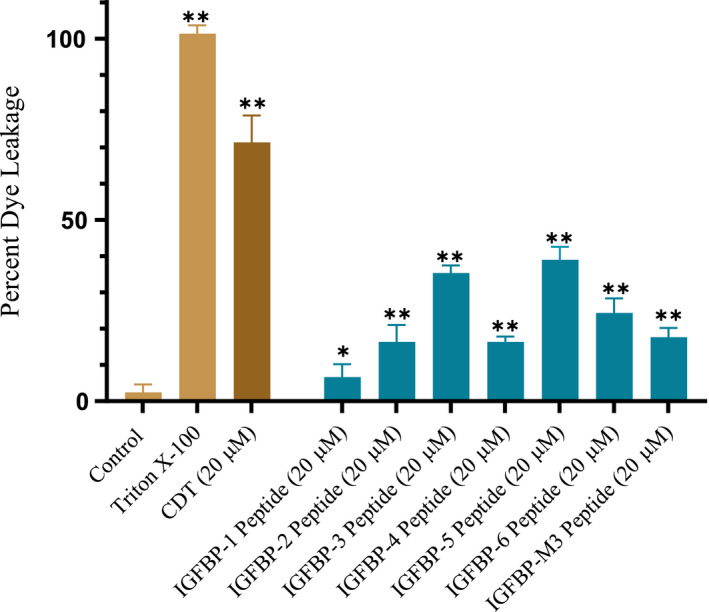
The IGFBP‐3 peptide has less liposome disruption capability compared to the CDT control. Liposomes were prepared as described in Materials and methods followed by addition of the IGFBP peptides. Negative controls contained 5% DMSO/PBS, which was predetermined not to cause dye leakage, while Triton X‐100 and CDT were used as positive controls. After a 10‐min period, fluorescence values of the samples in 96‐well plates were measured by a spectrofluorometer (filter set to 485 nm excitation and 528 nm emission). Fluorescence measurements indicate dye leakage corresponding to liposome damage. Triton X‐100 detergent (10% v/v in PBS) was used as the positive control for determination of 100% leakage. Percent liposome dye leakage was calculated, and each column represents the mean ± SD of three independent experiments, each run in triplicate. The asterisks (*P < 0.05, **P < 0.01) indicate a statistically significant difference from the control. The absence of asterisks indicates no significance, Mann–Whitney test.

Briefly, carboxyfluorescein‐encapsulated vesicles were made using a 3 : 1 mixture of 1‐palmitoyl‐2‐oleoyl‐glycero‐3‐phosphocholine (POPC)/1‐Palmitoyl‐2‐oleoyl‐sn‐glycero‐3‐(phospho‐rac‐(1‐glycerol)) (POPG) so that the model membrane would be 25% negatively charged, similar to that found in cancer cells [67]. The peptides (20 μm) were added to determine the relative extent of fluorescent dye leaked from the vesicles. Increased fluorescence due to leakage of the dye from the liposomes is indicative of the peptide efficiency to disrupt liposomes and, by extension, membranes. Liposome disruption might occur via pore formation or due to general solubilization of lipids in a manner similar to that of detergents [70]. Negative controls contained 5% DMSO/PBS and no peptide, while the positive controls (Fig. 1) included Triton X‐100 and cysteine‐deleted tachyplesin (CDT), shown previously to possess an approximate 70–78% membranolytic activity in the same liposomes at this concentration [67, 71, 72, 73].

Compared to CDT, the IGFBP peptides displayed mild‐to‐moderate membranolytic activity in the liposome dye leakage assay. Except for mutant IGFBP‐3, the membrane lytic activity and percent dye leakage appeared to correlate with the degree of overall positive charge (Table 1, Fig. 1). One explanation might be that peptides with a greater degree of positive charge are better attracted to the negatively charged liposomes. IGFBP‐3, IGFBP‐5, and IGFBP‐6 caused ~ 35%, ~ 39%, and ~ 24% dye leakage, respectively (Fig. 1). Charge alone is unlikely to account for these findings, however, since the mutant IGFBP‐3 peptide with a charge of +9 caused only ~ 17% dye leakage as compared to ~ 35% found for the WT IGFBP‐3 peptide (charge of +11) and ~ 24% for the IGFBP‐6 peptide with a charge of +6. This might point to K228 and R230 as important amino acids for the modest membranolytic activity of the IGFBP‐3 peptide.

The degree of liposome damage is less than that reported for membrane‐active peptides which tend to have alternating regions of positive charge and hydrophobicity [67, 71, 72, 73], however. The membrane lytic activities of the IGFBP‐3 and IGFBP‐5 peptides and their ability to disrupt the synthetic liposomes were lower (~ 35–39%) relative to that found for CDT (~ 72%) (Fig. 1). These results suggest that the membranolytic activity is unlikely to be the main mechanism by which the IGFBP‐3 peptide exhibits its cytotoxic effects.

### Stereochemistry is important for the wild‐type IGFBP‐3 peptide to both bind HA and to block viability of A549 cells expressing CD44

We next examined whether the binding of the IGFBP‐3 peptide to HA and its cytotoxic effects are stereochemistry‐dependent. *In vitro* cell culture studies have shown that d‐ and l‐peptides can either have identical performance in cell culture cytotoxicity assays or that the d‐peptide derivatives are at times found to be less cytotoxic than their l‐analogues, while on the other hand, the l‐enantiomer can have lower cytotoxicity than its d‐counterparts [74].

**Fig. 2:**
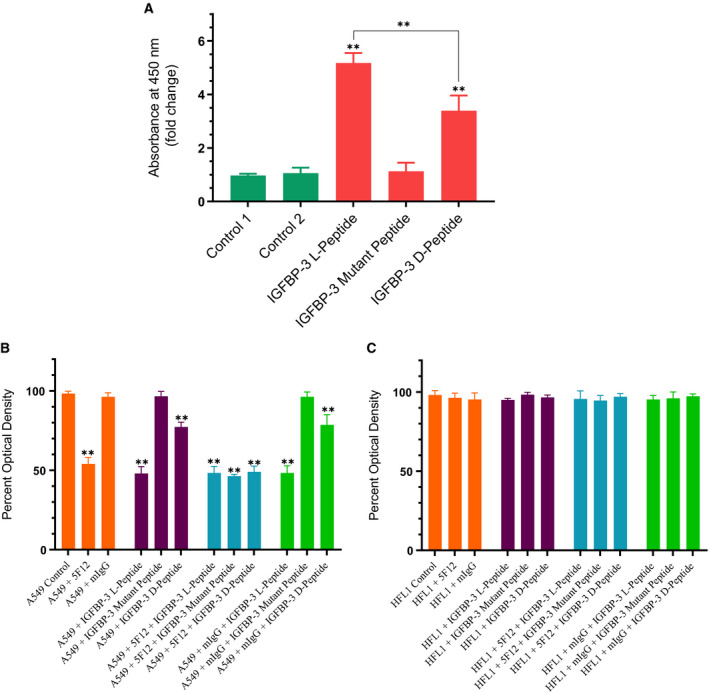
WT IGFBP‐3 l‐peptide is more effective than the d‐peptide in both binding HA and in blocking viability of A549 cells that express CD44 as compared to the CD44‐negative cell line, HFL1. (A) IGFBP‐3 peptides (50 nm each) were bound to the ELISA plate wells, and then, 200 nm biotin‐HA was added and processed as described in the Materials and methods section. The data were normalized to the control incubated with BSA (control 1), and fold change relative to the control was calculated. Control 2 is a negative control that included bound WT IGFBP‐3 l‐peptide and added streptavidin–HRP and TMB without addition of biotin‐HA. Each column represents the mean ± SD of three independent experiments, each run in triplicate. The asterisks (**P < 0.01) indicate a statistically significant difference from control 1 and of the IGFBP‐3 l‐peptide compared to the d‐counterpart. The absence of asterisks indicates no significance, Mann–Whitney test. (B, C) IGFBP‐3 peptides were added to cells in the absence or presence of the mIgG (5 μg·mL^−1^) antibody control or the CD44, 5F12 antibody (5 μg·mL^−1^), known to block HA‐CD44 interactions. Cell viability was assessed by the MTT assay. Cells were seeded in 96‐well plates at 0.2 × 10^5^ cells per well in 10% FBS‐supplemented media. The following day, the cell monolayers were incubated in serum‐free medium for 12 h and then treated as indicated for 48 h with the media containing the specific components in the different treatments replaced every 12 h. The concentration of IGFBP‐3 peptides added was 50 nm. The mIgG and CD44 antibodies were added either separately or 2 h prior to addition of IGFBP‐3 peptides. Optical density measurements (570 nm) were normalized by expressing each point in relation to the untreated control of each cell line (set to 100%). Each column represents the mean ± SD of three independent experiments, each run in triplicate. Asterisks (*) indicate a statistically significant difference from the corresponding untreated cell line control, *P < 0.05, **P < 0.01 of each cell line. The absence of asterisks indicates no significance, Mann–Whitney test.

IGFBP‐3 peptides (50 nm each) were bound to the ELISA plate wells; then, 200 nm biotin‐HA was added and processed as described in the Materials and methods section (Fig. 2A). The data were normalized to the control incubated with BSA, and fold change relative to the control was calculated (Fig. 2A). Each column represents the mean ± SD of three independent experiments, each performed in triplicate.

As expected and as we previously found [66], minimal binding to HA was observed upon using the IGFBP‐3 mutant peptide (Table 1, Fig. 2A). The binding of HA to the IGFBP‐3 d‐peptide was approximately 1.5‐fold lower compared to that of the l‐peptide. These results suggest that stereochemistry is an important factor for the interaction between the IGFBP‐3 peptide and HA.

We next compared the effect of the IGFBP‐3 d‐peptide on the viability of CD44‐negative normal human lung cells (HFL1) and human lung cancer cells (A549) known to express CD44 [66, 75]. Cells were seeded in 96‐well plates at 0.2 × 10^5^ cells per well in 10% FBS‐supplemented media. The following day, the cell monolayers were incubated in serum‐free medium for 12 h and then treated as indicated for 48 h with the media containing the specific components in the different treatments replaced every 12 h (Fig. 2B,C). IGFBP‐3 peptides (50 nm) were added to cells in the absence or presence of the mouse IgG isotype control with no relevant specificity to a target antigen (mIgG, 5 μg·mL^−1^) and the CD44 antibody (5F12, 5 μg·mL^−1^) known to block HA‐CD44 interactions [20, 41, 76]. We have previously used the 5F12 antibody to show that IGFBP‐3 binds HA and blocks its interactions with CD44 [48, 66]. The CD44 antibody was added either separately or 2 h prior to addition of IGFBP‐3 peptides. Cell viability was assessed by the MTT assay. Optical density measurements (570 nm) were normalized by expressing each point in relation to the untreated control of each cell line (set to 100%). Each column represents the mean ± SD of three independent experiments.

Since we previously found [48] that both the IGFBP‐3 protein and its WT peptide bind HA with comparable affinities, we examined the effect of the WT l‐peptide on cell viability relative to its d‐counterpart (Fig. 2B,C). Treatment of HFL1 cells with the antibody, 5F12, had little effect on cell viability (Fig. 2C). This finding was not surprising since HFL1 cells are known to express very little or no CD44 receptor [75]. The effects of the IGFBP‐3 l‐peptide on HFL1 were comparable to those of the D‐IGFBP‐3 and mutant peptides (Fig. 2C). This is likely due to the lack of CD44 expression in these cells since incubation with both the peptides and the antibody, 5F12, had only a minor effect compared to treatment with either the peptides or the antibody.

Consistent with our previous results [48], we found that the IGFBP‐3 l‐peptide but not its mutant reduces the viability of A549 cells, known to express CD44, by disrupting HA‐CD44 interactions (Fig. 2B). Blocking HA‐CD44 interactions in A549 cells with 5F12 reduced cell viability to the same extent as that found by the addition of only the IGFBP‐3 peptide or in combination (Fig. 2B). The IGFBP‐3 mutant peptide alone, with an impaired HA‐binding motif (Table 1), had little effect on A549 cell viability compared to the IGFBP‐3 l‐peptide (Fig. 2B), suggesting that binding HA is a prerequisite for the IGFBP‐3 peptide to exert its effects on cell viability.

Another important parameter seems to be the stereochemistry of the peptide since incubation of A549 cells with the D‐IGFBP‐3 peptide was not as efficient at reducing A549 cell viability as compared to the IGFBP‐3 l‐peptide (Fig. 2B), findings that might be accounted for by the reduced binding of the d‐peptide to HA (Fig. 2A). These results point to stereochemistry as an important factor in the biological performance of the IGFBP‐3 peptide and suggest that both the identity of the amino acids in a sequence and their stereochemistry are important determinants of the cytotoxic functions of the IGFBP‐3 peptide.

### IGFBP‐3 N‐glycosylation or reduction does not alter its ability to interact with either HA or HN

**Fig. 3:**
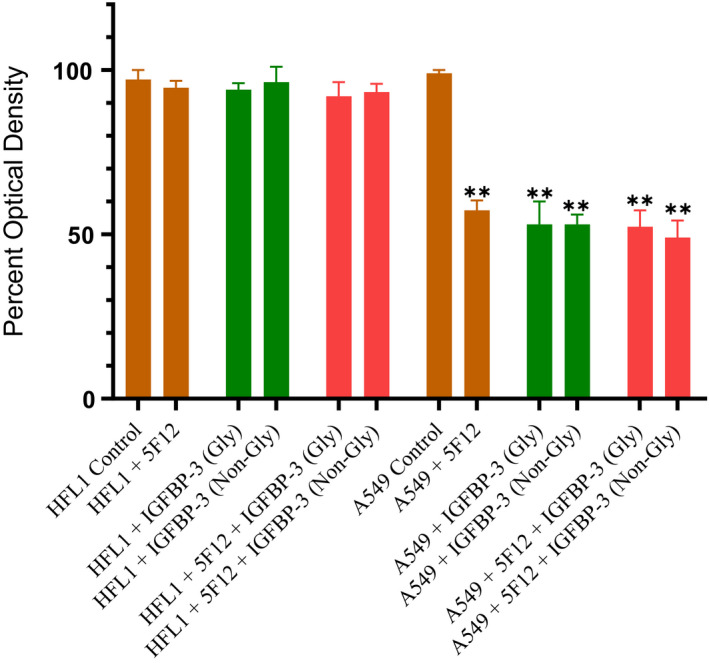
A comparable decrease in A549 cell viability is observed upon using either glycosylated or nonglycosylated IGFBP‐3 protein. IGFBP‐3 protein glycosylated (Gly) or nonglycosylated (Non‐Gly) was added (50 nm) to cells in the absence or presence of the CD44 antibody, 5F12, known to block HA‐CD44 interactions. Cell viability was assessed by the MTT assay. Cells were seeded in 96‐well plates at 0.2 × 10^5^ cells per well in 10% FBS‐supplemented media. The following day, the cell monolayers were incubated in serum‐free medium for 12 h and then treated as indicated for 48 h with the media containing the specific components in the different treatments replaced every 12 h. The CD44 antibody (5 μg·mL^−1^) was added either separately or 2 h prior to addition of IGFBP‐3 proteins. Optical density measurements (570 nm) were normalized by expressing each point in relation to the untreated control of each cell line (set to 100%). Each column represents the mean ± SD of three independent experiments, each performed in triplicate. Asterisks (*) indicate a statistically significant difference from the corresponding untreated cell line control, *P < 0.05, **P < 0.01 of each cell line. The absence of asterisks indicates no significance, Mann–Whitney test.

Asn89, Asn109, and Asn172 have been identified as the specific sites of N‐linked glycosylation in the N‐terminal and linker domains of IGFBP‐3 [5]. These amino acid residues are not located in the IGFBP‐3 peptide synthesized in this study that spans residues 215–232, known to contain the binding regions for both HN [55, 56] and HA [1, 48, 49, 62, 66]. We therefore elected to use the full‐length IGFBP‐3 protein to examine the effect of glycosylation, if any, on cell viability and the ability of the protein to bind HA or HN. The effect of glycosylated and nonglycosylated IGFBP‐3 (Materials and methods) on HFL1 and A549 cell viability was assessed by the MTT assay (Fig. 3). Cells were seeded in 96‐well plates at 0.2 × 10^5^ cells per well in 10% FBS‐supplemented media. After 24 h, the cells were incubated in serum‐free medium for 12 h and then treated as indicated for 48 h with the media containing the specific components in the different treatments replaced every 12 h. The glycosylated or nonglycosylated IGFBP‐3 protein (50 nm) was added to cells in the absence or presence of the CD44 antibody, 5F12, known to block HA‐CD44 interactions. The CD44 antibody (5 μg·mL^−1^) was added either separately or 2 h prior to addition of the IGFBP‐3 proteins. Optical density measurements (570 nm) were normalized by expressing each point in relation to the untreated control of each cell line (set to 100%). Each column represents the mean ± SD of three independent experiments, each carried out in triplicate (Fig. 3).

Compared to the lack of effect observed using the CD44‐negative cell line, HFL1, a comparable decrease in A549 cell viability was found upon using either glycosylated or nonglycosylated IGFBP‐3 protein. While we have no data to suggest that there are conformational changes of the IGFBP‐3 protein upon glycosylation, the lack of effect on cell viability upon glycosylation of Asn89, Asn109, and Asn172 suggests that changes in protein conformation, if any, are not important for the cytotoxic effects of IGFBP‐3.

We previously found that the 18‐amino acid residue peptide (^215^‐KKGFYKKKQCRPSKGRKR‐^232^) is able to bind HA with comparable affinity as that found for the full‐length IGFBP‐3 protein and that the HA‐binding site is largely contained within this basic region of the protein [48, 66]. To disrupt the HA‐binding motif and to identify amino acid residues important for binding HA, we synthesized the K228AR230A mutant peptide and found that these mutations completely abolished the peptide's ability to bind HA [66].

**Fig. 4:**
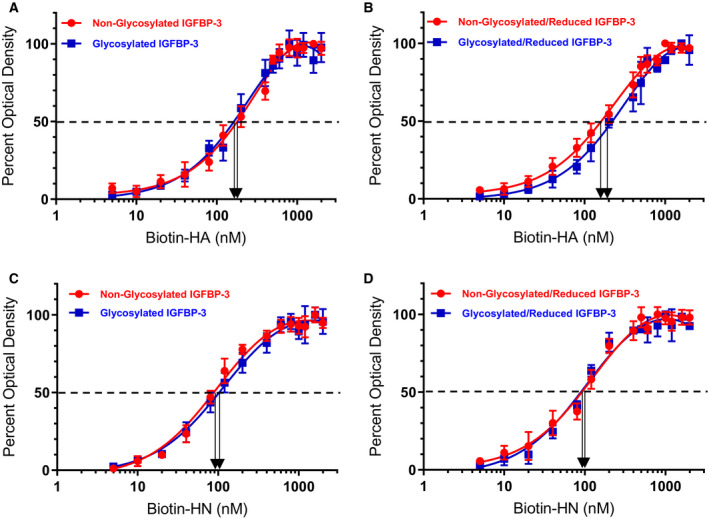
Binding of biotin‐HN or biotin‐HA to IGFBP‐3 is not altered by IGFBP‐3 glycosylation. Binding of biotin‐HN or biotin‐HA to immobilized glycosylated or nonglycosylated IGFBP‐3 was measured by ELISA. IGFBP‐3 (50 nm) was bound to the wells; then, increasing concentrations of biotin‐HN or biotin‐HA were added and processed as described in the Materials and methods. The curves were drawn using the graphpad prism 8.4.2 software. Data were expressed as the mean ± SD of three independent experiments.

To examine whether glycosylation or reduction of IGFBP‐3 alters the ability of the protein to bind either HN or HA, *in vitro*, glycosylated or nonglycosylated IGFBP‐3 reduced or nonreduced (50 nm, Materials and methods) was absorbed to microtiter plate wells and allowed to bind overnight at 4 °C. Biotin‐HN or biotin‐HA was then added at concentrations ranging from 0 to 2000 nm and incubated in triplicate wells (one experiment) overnight at 4 °C (Table 2, Fig. 4). Following methods we previously used [48, 60, 66, 68], to monitor nonspecific binding, negative control wells on the plates included bound IGFBP‐3 then adding streptavidin–horseradish peroxidase and TMB without addition of biotin‐HN or biotin‐HA.

**Table 2:** Effect of IGFBP‐3 modifications on its binding to HA or HN. IGFBP‐3 (50 nm) was bound to the ELISA plate wells, and then, 0–2 μm biotin‐HA or biotin‐HN was added and processed as described in the Materials and methods section. The blank containing all components but without biotin‐HA or biotin‐HN was subtracted from all values; then, the optical density was normalized by expressing each point in relation to the best‐fitted Emax value (set to 100%). The concentration of biotin‐HA or biotin‐HN (nm) that produced 50% of maximum binding is shown as the mean ± SD of three independent experiments, each run in triplicate. Controls included samples that were alkylated without prior reduction.

	IGFBP‐3 Nonglycosylated	IGFBP‐3 Glycosylated	IGFBP‐3 Nonglycosylated/Reduced	IGFBP‐3 Glycosylated/Reduced
Biotin‐HA	182 ± 32	180 ± 28	176 ± 26	190 ± 28
Biotin‐HN	92 ± 16	104 ± 14	96 ± 12	98 ± 16

None of the modifications had an effect on the binding of IGFBP‐3 to HN or HA (Table 2, Fig. 4). None of the residues known to be glycosylated in IGFBP‐3 (Asn89, Asn109, and Asn172) are located in the IGFBP‐3 C‐terminal region containing amino acid residues, 215–232, and only cysteine 224 is found in this 18‐amino acid basic motif. Therefore, no residues are known to be glycosylated in this region and no disulfide bonds can be formed. Similarly, HN was also found to bind the 215–232 residue peptide in a manner similar to that of the full‐length protein [48, 66]. The finding that glycosylation and reduction do not alter the binding of IGFBP‐3 to either HA or HN suggests that the basic 18‐amino acid residue sequence of IGFBP‐3 remains accessible to either HN or HA upon glycosylation or reduction of the full‐length protein.

### Both deglycosylation and disulfide bond formation are important for the ability of CD44 to compete with IGFBP‐3 for binding HA

**Fig. 5:**
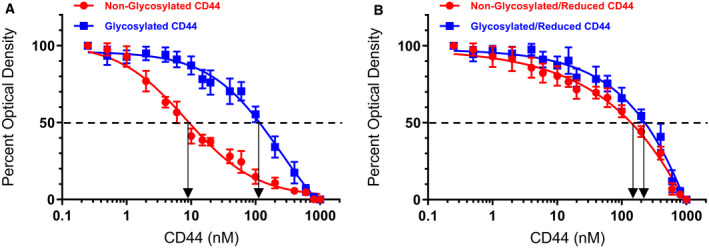
Deglycosylation and disulfide bonds increase the ability of CD44 to compete with IGFBP‐3 for binding biotin‐HA. IGFBP‐3 (10 nm) was bound to the ELISA plate wells. Biotin‐HA (100 nm) was then added along with increasing concentrations of CD44. The curves were drawn using the graphpad prism 8.4.2 software. Data were expressed as the mean ± SD of three independent experiments, each run in triplicate. Arrows on the *x*‐axis indicate the CD44 concentration that corresponds to 50% inhibition for each curve. The dashed line indicates 50% of maximum binding.

Previous reports have shown that the strength of the HA‐CD44 interaction is modulated by attachment of carbohydrates to O‐ and N‐linked glycosylation sites on the extracellular portion of CD44 that includes the HA‐binding domain, HABD [20, 21, 33, 37, 41]. While inhibition of O‐glycosylation did not have a significant effect on the frequency of binding of either CD44v or CD44s to HA [19], N‐linked glycosylation was shown to positively correlate with binding of CD44 to HA in lung epithelium‐derived tumor cells expressing CD44s which include the A549 cells used in this study [21, 43]. We, therefore, tested the effect of removing N‐linked oligosaccharides from CD44, upon PNGase F treatment (Materials and methods), on its ability to compete with IGFBP‐3 for binding HA (Table 3, Fig. 5). Since neither glycosylation nor reduction appeared to affect the binding of IGFBP‐3 to HA (Table 2, Fig. 4A,B), glycosylated IGFBP‐3 (10 nm) was bound to the ELISA plate wells (Table 3, Fig. 5). A single concentration of biotin‐HA (100 nm), according to methods we used earlier [48], was incubated for 1 h with varying concentrations of glycosylated or nonglycosylated CD44 (0–1000 nm) before loading into the IGFBP‐3‐coated wells (Table 3, Fig. 5). Streptavidin–horseradish peroxidase was then added at the manufacturer's recommendation and incubated for 3 h at RT, and the color was developed as described in the Materials and methods section.

**Table 3:** Effect of CD44 modifications on its competition with IGFBP‐3 for binding HA. IGFBP‐3 (10 nm) was bound to ELISA plate wells. Biotin‐HA (100 nm) was then added with increasing concentrations of CD44 and processed as described in the Materials and methods section. The blank containing all components but without biotin‐HA was subtracted from all values; then, the optical density was normalized by expressing each point in relation to the best‐fitted Emax value (set to 100%). The concentration of CD44 (nm) that produced 50% of maximum binding is shown as the mean ± SD of three independent experiments, each run in triplicate. Controls included samples that were alkylated without prior reduction.

	CD44 Nonglycosylated	CD44 Glycosylated	CD44 Nonglycosylated/Reduced	CD44 Glycosylated/Reduced
Biotin‐HA	9 ± 2	128 ± 24	176 ± 37	210 ± 41

The results showed that nonglycosylated CD44 was more efficient at inhibiting the binding of biotin‐HA to immobilized IGFBP‐3 than glycosylated CD44 (Table 3, Fig. 5). The concentration of CD44 indicated by the arrows on the *x*‐axis (Fig. 5) that corresponded to 50% inhibition was found to be 9 ± 2 nm for nonglycosylated CD44. This value increased to 128 ± 24 nm upon using the glycosylated protein, suggesting that removing the N‐linked oligosaccharides strengthened the ability of CD44 to compete with IGFBP‐3 for binding HA.

We next tested whether reduction of CD44 affects its ability to compete with IGFBP‐3 for binding HA. It was previously found that CD44 activity is regulated by the extracellular redox environment via the secretion of thiol reductase and protein disulfide isomerase [45] enzymes. A labile disulfide bond formed by Cys77 and Cys97 in CD44 was shown to be important for stabilizing the HA‐binding groove [47]. Reduction of this bond inhibited HA‐CD44 interaction *in vitro*, and the ability of cells to adhere to HA‐coated surfaces was blocked by pretreatment with reducing agents [47]. Moreover, reduction of recombinant soluble CD44 was found to inhibit its ability to bind HA [47].

ELISA binding assays were carried out as described for glycosylated and nonglycosylated CD44 (Table 3, Fig. 5). Our results show that CD44 was relatively ineffective at blocking HA‐IGFBP‐3 interactions when reduced (Table 3, Fig. 5). The concentration of CD44 (nonglycosylated/reduced) that resulted in 50% inhibition of the binding of biotin‐HA to IGFBP‐3 was 176 ± 37 as compared to 9 ± 2 for CD44 (nonglycosylated) (Table 3, Fig. 5). Of all the treatments, the highest CD44 concentration (210 ± 41 nm) needed to result in 50% inhibition of the binding between biotin‐HA and IGFBP‐3 bound to the wells was that of the glycosylated and reduced protein (Table 3, Fig. 5), suggesting that both deglycosylation and disulfide bond formation are important for the ability of CD44 to compete with IGFBP‐3 for binding HA.

## Conclusion

In this study, we examined properties that might be important for the interaction of IGFBP‐3 with HA. The C‐terminal domain of IGFBP‐3 is known to contain an 18‐basic amino acid motif defined by amino acid residues 215–232 of mature IGFBP‐3, previously shown to bind heparin and certain glycosaminoglycans, including HA [1, 6, 49, 50, 51]. Using an *in vitro* assay, as we reported previously [67] where fluorescence measurements indicate dye leakage corresponding to membrane damage, we tested whether the synthetic cationic 18 residue peptide (Table 1) possesses physiochemical features with membrane lytic properties. Relative to CDT, shown previously to have an approximate 70–78% membranolytic activity [67, 71, 72, 73], the IGFBP‐3 peptide only exhibited mild‐to‐moderate activity in the liposome dye leakage assay (Fig. 1). These findings might rule out the membranolytic activity as a main mechanism used by IGFBP‐3 to induce its effects.

d‐peptides and l‐peptides can either exhibit their effects in an identical manner in cell culture cytotoxicity assays, or d‐peptide derivatives can be more or less cytotoxic than their stereochemically opposite l‐counterparts [74]. The IGFBP‐3 d‐peptide was synthesized in order to examine whether the binding of the IGFBP‐3 peptide to HA and its cytotoxic effects are dependent upon stereochemistry. The IGFBP‐3 d‐peptide bound HA with an approximate 1.5‐fold decrease compared to the l‐peptide (Fig. 2A). These results suggest that stereochemistry is an important factor for the interaction between the IGFBP‐3 peptide and HA. Since we found earlier [48] that both the IGFBP‐3 protein and its WT peptide bind HA with comparable affinities, we tested the effect of the WT L‐peptide on cell viability relative to its D‐counterpart (Fig. 2B,C). Using the CD44‐negative normal human lung cells (HFL1) and human lung cancer cells (A549) known to express CD44 [66, 75], we found that the IGFBP‐3 l‐peptide, but not its mutant, reduces A549 cell viability by disrupting HA‐CD44 interactions (Fig. 2B,C), a result consistent with our previous reports [48]. Treatment of A549 cells with the IGFBP‐3 d‐peptide, however, reduced cell viability by only ~ 23%, compared to the reduction of ~ 52% obtained with the IGFBP‐3 l‐peptide (Fig. 2B,C). These findings might be accounted for by the reduced binding of the IGFBP‐3 d‐peptide to HA (Fig. 2A). These results show that stereochemistry, along with the identity of the amino acid residues, is an important determinant of the binding of the IGFBP‐3 peptide to HA and for execution of its biological performance.

**Fig. 6:**
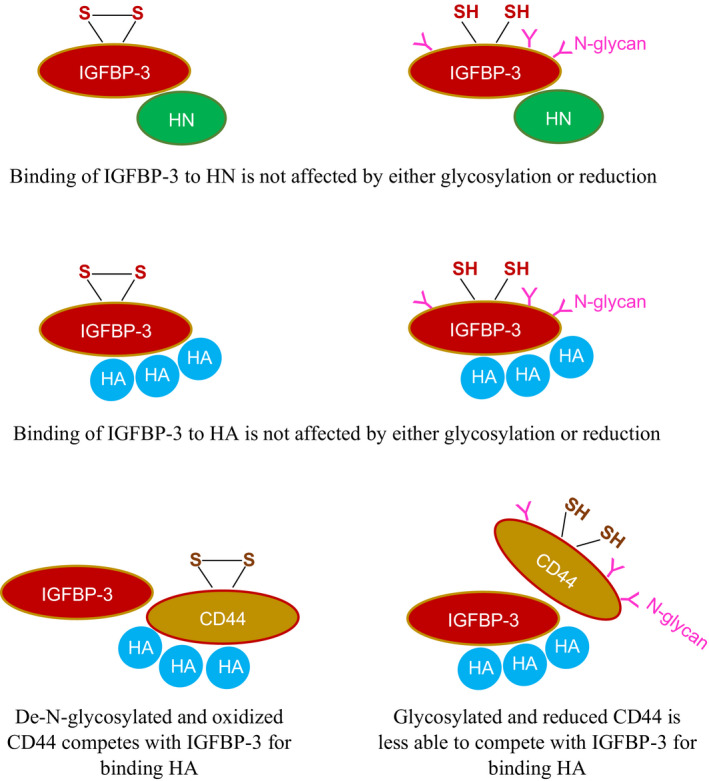
Schematic representation of the main findings of this study. Binding of IGFBP‐3 to either humanin (HN) or to the glycosaminoglycan, hyaluronan (HA), is not affected by either glycosylation or reduction. Glycosylated and reduced CD44 is less able than de‐N‐glycosylated and oxidized CD44 to compete with IGFBP‐3 for binding HA.

IGFBP‐3 is known to be glycosylated on Asn89, Asn109, and Asn172 in the N‐terminal and linker domains [5]. These amino acid residues are not located in the IGFBP‐3 segment composed of the 18‐amino acid basic motif defined by residues 215–232 and known to contain the binding regions for both HN [55, 56] and HA [1, 48, 49, 62, 66]. The decrease in A549 cell viability was not altered upon treatment with either the glycosylated or nonglycosylated IGFBP‐3 protein (Fig. 3). Furthermore, binding of IGFBP‐3 to either HA or HN was unaffected by glycosylation or reduction of the nine disulfide bonds, six of which located in the N‐terminal domain and three in the C‐terminal domain [1, 5] (Table 2, Fig. 4). Only cysteine 224 is located in the 18‐amino acid basic motif of the protein (Table 1). Therefore, no residues are known to be glycosylated in this region and no disulfide bonds can be formed. We previously showed that the 18‐amino acid basic peptide is able to bind HA or HN with comparable affinities to that found for the full‐length IGFBP‐3 protein, *in vitro*, and that the HA‐binding site is predominantly contained within this basic region of the protein [48, 66]. Thus, our data might suggest that the basic 18‐amino acid residue sequence (^215^‐KKGFYKKKQCRPSKGRKR‐^232^) of IGFBP‐3 remains accessible for interaction with either HN or HA upon glycosylation or reduction of the full‐length protein (Fig. 6).

The strength of HA‐CD44 interaction is known to be modulated by attachment of carbohydrates to O‐ and N‐linked glycosylation sites on the extracellular portion of CD44 that include HABD, the HA‐binding domain, [20, 21, 33, 37, 41]. N‐linked glycosylation was found earlier to positively correlate with binding of CD44 to HA in lung epithelium‐derived tumor cells expressing CD44s which include the A549 cells used in this study [21, 43]. Inhibition of O‐glycosylation did not significantly affect the frequency of binding of either CD44v or CD44s to HA [19]. Consistent with previous reports [42], removing N‐linked oligosaccharides from CD44 by treatment with PNGase F increased its ability to compete with IGFBP‐3 for binding HA (Table 3, Fig. 5). Previous studies showed that reduction of a labile Cys77–Cys97 disulfide bond in CD44 blocked HA‐CD44 interaction *in vitro* and the ability of cells to adhere to HA‐coated surfaces [47]. In accord with these studies, our results show that CD44 was relatively ineffective at blocking HA‐IGFBP‐3 interactions when reduced (Table 3, Fig. 5), suggesting that both deglycosylation and disulfide bond formation are important for the ability of CD44 to compete with IGFBP‐3 for binding HA (Fig. 6).

How signaling between IGFBP‐3 and other molecular players, such as IGFBP‐7, might modulate its ability to curb proliferation and induce apoptosis merits further investigation. Our laboratory is currently probing into regulation of the interactions between IGFBP‐3 and HA and consequent outcome on HA‐CD44 signaling in cell lines expressing different levels of CD44 and IGFBP‐3.

## Conflict of interest

All authors read and approved the final manuscript and declare no conflict of interest with the contents of this article. ‘The content is solely the responsibility of the authors and does not necessarily represent the official views of the National Institutes of Health’.

## Author contributions

All authors have given approval to the final version of the manuscript. HGE designed and coordinated the study, supervised the project, and wrote the paper. SD performed ELISA binding kinetics, deglycosylation, reduction, and cell viability assays. JD performed the liposome dye leakage assays. BC, JD, and DP performed the peptide synthesis and purification under the supervision of DLH, RM and DP helped with the cell viability assays and ELISAs. JG maintained the cells and provided advice on tissue culture. All authors read and approved the final manuscript.

## Data Availability

The original data are available upon reasonable request.
